# Transcriptome-Wide Cox Regression Identifies Candidate Survival-Associated Genes in Newly Diagnosed Acute Myeloid Leukemia

**DOI:** 10.1177/11769351261478087

**Published:** 2026-08-08

**Authors:** Songphol Tungjitviboonkun, Nikhil Mullapudi, Elly Gardev

**Affiliations:** 1 Department of Epidemiology & Biostatistics, 8785 University of California San Francisco, San Francisco, CA, USA

**Keywords:** acute myeloid leukemia, gene expression, Cox regression, transcriptome-wide analysis, biomarker

## Abstract

**Objectives:**

Acute myeloid leukemia (AML) exhibits substantial biological heterogeneity that is not fully captured by current prognostic systems. We aimed to identify transcriptome-wide gene expression markers associated with overall survival in newly diagnosed AML patients.

**Methods:**

Gene expression and clinical data from 399 newly diagnosed AML patients in the OHSU cohort were obtained via cBioPortal. Cox proportional hazards regression was performed independently for 22,836 genes to evaluate associations with overall survival. Analyses were restricted to genes with sufficient observations, and Bonferroni correction was applied to account for multiple testing.

**Results:**

A total of 46 genes were significantly associated with overall survival after multiple testing correction. Higher expression of *FAM213A* (hazard ratio [HR] 1.240) and *TJP2* (HR 1.439) was associated with increased mortality risk, whereas higher expression of *PJA2* (HR 0.381), *MICALL2* (HR 0.779), and *LTK* (HR 0.828) was associated with improved survival. Several identified genes, including *FAM213A, MICALL2*, and *PJA2*, have known roles in cancer biology, supporting the biological plausibility of these findings.

**Conclusions:**

This transcriptome-wide analysis identified multiple genes associated with survival in newly diagnosed AML, highlighting candidate prognostic biomarkers that may complement existing risk stratification frameworks. Further validation in independent cohorts is warranted.

## Description

Acute myeloid leukemia (AML) is a heterogeneous hematologic malignancy characterized by clonal proliferation of abnormal myeloid cells. Although cytogenetic and molecular risk stratification are widely used for prognosis, they do not fully capture transcriptional heterogeneity that may influence clinical outcomes. We performed a transcriptome-wide survival analysis to identify gene expression patterns associated with overall survival in newly diagnosed AML.

Gene expression and clinical data were obtained from the AML (OHSU) dataset available through cBioPortal. To reduce confounding from disease progression and treatment resistance, the analysis was restricted to newly diagnosed patients, yielding a study population of 399 individuals. When multiple samples were available per patient, only the first sample was included. Gene expression data were merged with clinical information using unique patient identifiers. Overall survival, measured in months, was the primary outcome.

A Cox proportional hazards regression model was fitted independently for each of 22,836 genes, with gene expression as the predictor and survival time as the outcome. Analyses were restricted to genes with more than three observations to ensure model stability. Bonferroni correction was applied to account for multiple comparisons, and statistical significance was defined as a corrected P value threshold.

**Figure 1.**
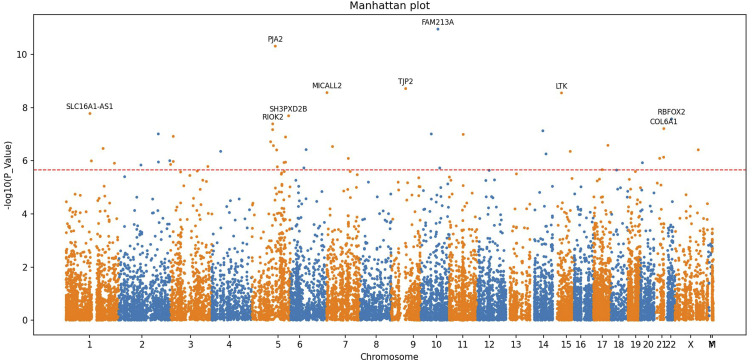
Manhattan plot of transcriptome-wide Cox regression results for overall survival in newly diagnosed AML.

A total of 46 genes were significantly associated with overall survival. The top-ranked genes are summarized (Table 1). Among these, higher expression of *FAM213A* (hazard ratio [HR] 1.240) and *TJP2* (HR 1.439) was associated with increased hazard of death, suggesting poorer prognosis. In contrast, higher expression of *PJA2* (HR 0.381), *MICALL2* (HR 0.779), and *LTK* (HR 0.828) was associated with reduced mortality risk and improved survival. A Manhattan plot illustrating the transcriptome-wide results is shown (Figure 1). These findings indicate that variation in gene expression may reflect clinically relevant differences in AML outcomes.

**Table 1.** Top Genes Associated With Aggressive AML

Gene	HR	95% CI	P-value	CHR	Starting bp	Ending bp
FAM213A	1.240	1.165 to 1.319	1.123 x 10^-11^	10	80406443	80437115
PJA2	0.381	0.285 to 0.508	4.887 x 10^-11^	5	109334704	109409991
TJP2	1.439	1.278 to 1.620	1.927 x 10^-9^	9	69121264	69274615
MICALL2	0.779	0.718 to 0.846	2.750 x 10^-9^	7	1428465	1459496
LTK	0.828	0.778 to 0.881	2.822 x 10^-9^	15	41503634	41513887
SLC16A1-AS1	1.438	1.268 to 1.632	1.677 x 10^-8^	1	112849483	113047059
SH3PXD2B	1.228	1.143 to 1.319	2.051 x 10^-8^	5	172325000	172454531
RBFOX2	1.207	1.129 to 1.289	2.675 x 10^-8^	22	35738736	36028824
RIOK2	0.422	0.310 to 0.575	4.125 x 10^-8^	5	97160867	97183289
COL6A1	1.169	1.105 to 1.237	6.186 x 10^-8^	21	45981755	46005050

**Abbreviations:** HR, hazard ratio; CI, confidence interval; CHR, chromosome; bp, base pairs; AML, acute myeloid leukemia. Genomic coordinates are based on GRCh38/hg38.

Several of the identified genes are biologically plausible. *FAM213A* has been implicated in oxidative stress regulation and myelopoiesis and has been linked to adverse prognosis in AML.^1^ *MICALL2* is an emerging oncogene associated with tumor progression in multiple cancers.^2-4^ Conversely, *PJA2* functions as an E3 ubiquitin ligase with tumor suppressor activity, and its downregulation has been associated with poor outcomes in solid tumors.^5,6^ These observations support the potential relevance of the identified genes in AML pathobiology.

This study has important limitations. First, the analysis was conducted in a single publicly available cohort, which may limit generalizability. Second, models were primarily univariable and did not adjust for established prognostic factors such as age, cytogenetic risk, treatment regimens, and mutational profiles. Therefore, the identified genes should be considered candidate markers rather than independent prognostic factors. Additionally, the use of stringent Bonferroni correction may have reduced sensitivity to detect genes with moderate effects. The study limits participants to newly-diagnosed AML patients to reduce confounding of chemoresistance-associated molecular mechanisms, yet emerging evidence highlights the relevance of such mechanisms and candidate therapeutic biomarkers in AML. Epitranscriptomic regulators, such as METTL3-mediated m6A RNA methylation, have been implicated in resistance to venetoclax and anthracyclines, and broader classes of leukemic stem cell, circulating, and molecular biomarkers have also been explored for their relevance to treatment response prediction.^7,8^ Integration of the transcriptome-wide markers identified here with such resistance-associated pathways may offer additional insight into AML prognosis and treatment response.

In summary, this transcriptome-wide Cox regression analysis identified multiple genes associated with overall survival in newly diagnosed AML. These findings provide a preliminary set of candidate biomarkers that may complement existing prognostic frameworks. Future studies should validate these results in independent cohorts and evaluate whether these genes retain prognostic significance after adjustment for known clinical and molecular factors, as well as formally assess predictive performance using time-dependent ROC/AUC analysis to establish clinical utility. The full analysis code used in this study is publicly available at: https://github.com/stvtung/TWAS-AML.

## Data Availability

The datasets analyzed during the current study are publicly available through the cBioPortal for Cancer Genomics database (https://www.cbioportal.org). The data used in this study are publicly accessible and can be obtained from the corresponding cBioPortal datasets.
